# Editorial: Women in science—Regulatory science 2021

**DOI:** 10.3389/fmed.2023.1159815

**Published:** 2023-03-16

**Authors:** Mette Due Theilade Thomsen, Lisbeth Ehlert Knudsen

**Affiliations:** ^1^PIP Adviser, Lyngby, Denmark; ^2^Department of Public Health, University of Copenhagen, Copenhagen, Denmark

**Keywords:** scientific advice, drug repurposing, pediatric drug development, drug registration, healthcare, funding, clinical research, gender research

## Introduction

All publications in this Research Topic on Women in Regulatory Science have female first authors, and the diversity in scientific subjects and high quality of the publications truly underline that regulatory science is blessed with a high number of very specialized, extremely skillful and high performing women. The publications indeed demonstrate how women are moving science forward.

## Scientific advice

Murphy et al. examine the contributions of patient participation in scientific advice procedures at the European Medicines Agency (EMA), describing methodology used to involve patients in scientific advice and presenting an analysis of feedback received from EMA procedure coordinators as well as patients who have participated. There is a significant added value from patient engagement in EMAs Scientific Advice procedures suggesting the need to further expand patient input to real-world evidence for the benefit of public health.

Dekker et al. address new approaches in the use of remote monitoring technologies (RMT) in clinical registration trials by evaluating regulatory qualification opinions, qualification and scientific advices provided between 2013 and 2019 by the EMA Committee for Medicinal Products for Human Use (CHMP). The RMTs included accelerometers to measure activity and/or sleep, mobile applications and glucose monitoring devices, mostly proposed as secondary or exploratory endpoints. CHMP recommendations concerned relevance, validation, precision, compliance and actual use as well as privacy and data handling. RMTs in registration trials are still rare but use has increased over time. This insight may stimulate the use of novel RMTs in a regulatory context.

## Repurposing of medicines

Drug repurposing is the process of identifying a new use for an existing medicine in an indication outside the scope of the original approved indication. Asker-Hagelberg et al. address the issue of repurposing of authorized medicines taking the examples collected during the COVID-19 pandemic into consideration and stressing the need for initiatives. A European Union framework for repurposing of established medicines is described.

## Pediatric drug development

The EU Pediatric Regulation was introduced in 2007 and is currently undergoing revision. A pediatric legislation has existed for even longer in the USA. Existing differences in the legislative framework may cause different pediatric requirements for similar indications granted for similar drugs across jurisdictions. In a cross-sectional study, Christiansen et al. study mandatory requirements for pediatric drug development in the EU and the US, comparing requirements for therapeutic indications granted at the time of initial approval for novel drugs approved in the two regions from 2010 to 2018. This is an important contribution to the evaluation of how aligned requirements for pediatric drug development are across the regions.

## Global drug registration requirements

Zhong et al. compared registration requirements to Proprietary Chinese medicine in Hong Kong and Canada based on publicly available information. Similarities and differences exist between the two regulatory systems in terms of quality, safety and efficacy requirements. Knowledge of the Proprietary Chinese Medicines product license application procedure and requirements in Hong Kong and Canada will enable an appropriate strategy for gaining product approval.

## General healthcare

Enticott et al. describe Australian experiences with a Learning Health System stressing the need for cross disciplinary work and data sharing. The study aimed to describe the process and present a perspective on a coproduced Learning Health System framework, with development led by publicly funded Academic Health Research Translation Centres with a mandate to integrate research into healthcare to deliver impact. This continuous learning approach aims to deliver evidence-based healthcare improvement.

## Funding and innovation

Diabetes Mellitus (DM) is one of the World Health Organization's priority diseases under research by the program of Innovative Medicines Initiative (IMI). Brito et al. reviewed the Impact of the IMI initiatives related to DM by analyzing publications from projects under the initiative. The IMI funded projects identified new biomarkers, medical and research tools, clinical trial designs, clinical endpoints and therapeutic targets, to name a few. Based on the scientific data produced, the authors provide a joint vision with strategies for integrating personalized medicine into healthcare practice.

Janssens et al. studied patient preferences for Multiple Myeloma Treatments by qualitative interviews in 4 EU countries and thematic analysis. Results pointed at the need for Multiple Myeloma drug development, evaluation and individual treatment not only focusing on extending the life but also taking side effects into account as these significantly impact Multiple Myeloma patients' quality of life.

Sessa et al. describe the role and limitations of the European Patients Academy on Therapeutic Innovation (EUPATI) in Switzerland (CH) in promoting patient involvement in medicines research and development. EUPATI CH initiated a multi-stakeholder survey involving patient representatives, academia, pharmaceutical industry, healthcare professionals, and government agencies. A need for collaboration amongst stakeholders as well as funding, knowledge and human resources was identified.

## Clinical development

Kearney et al. describe how various stakeholders can utilize regulatory affairs and clinical affairs to navigate the nuanced landscape behind the development and use of clinical diagnostic products. This work emphasizes the critical importance of utilizing regulatory affairs and clinical affairs as an integral part of product development to ensure sustained innovation.

Monti et al. stress the need for academic follow-up studies postmarketing identifying barriers and possible solutions from experiences with breast cancer. The authors describe the regulatory hurdles of getting approvals for an academic study funded by an EU call on validation of biomarkers for personalized cancer medicine.

We conclude this editorial with a gender-related research study. Gender medicine investigates the influence of sex/gender on the pathophysiology, prevention and treatment of disease, and on social and psychological aspects. Medical research was previously performed dominantly on men in preclinical and clinical studies, but the picture is changing. Artificial intelligence (AI) algorithms assist health professionals with data management, preclinical image-based diagnostics, robotic surgery, prediction models, and decision-making support. Yoon et al. conducted a bibliometric analysis of gender-related articles in medical AI over 20 years. The number of publications and percentage of gender-related articles in medical AI fields increased from 2001 to 2020, with a steep increase in the last 5 years. This underlines an increased focus on gender-related medical research, to the benefit of the patients.

## Author contributions

All authors contributed to the article and approved the submitted version.

